# Investigation of Treatment‐Related Disparities in Metastatic Pancreatic Cancer Patients Using Real‐World Data

**DOI:** 10.1002/cam4.71108

**Published:** 2025-08-07

**Authors:** Lavanya Aluri, Christina Brown, Anush Sridharan, Elizabeth Handorf, Kristen Sorice, Efrat Dotan, Shannon M. Lynch

**Affiliations:** ^1^ Lewis Katz School of Medicine Temple University Philadelphia Pennsylvania USA; ^2^ Department of Medical Oncology, Fox Chase Cancer Center Philadelphia Pennsylvania USA; ^3^ Cancer Prevention and Control, fox Chase Cancer Center Philadelphia Pennsylvania USA; ^4^ Center for Biostatistics and Epidemiology, Lewis Katz School of Medicine Temple University Philadelphia Pennsylvania USA

**Keywords:** health disparities, neighborhood, pancreatic cancer, social determinants, survival

## Abstract

**Background:**

Racial disparities exist in the overall treatment of patients with metastatic pancreatic adenocarcinoma (mPDAC). Compared to White patients, patients of color are less likely to undergo surgical resection for early‐stage disease and receive chemotherapy. This study explored the time to first treatment (T2FT), receipt of standard guideline‐concordant first‐line therapies, and overall survival in an mPDAC population by race/ethnicity.

**Methods:**

Utilizing real‐world United States data from the Flatiron Health electronic health record (EHR) derived, deidentified database, T2FT was defined as the difference between the diagnosis date of mPDAC and the initiation of first‐line systemic anticancer therapy in days; receipt of standard‐guideline concordant first‐line therapy was defined based on the National Comprehensive Cancer Network guidelines. We assessed disparities in T2FT and overall survival by race using Kaplan–Meier curves with log‐rank tests and Cox proportional hazards regression models.

**Results:**

In the study population of interest, there was a significant difference in T2FT for Black compared to White patients. This effect is attributable to the first 2 months following diagnosis. There was no evidence of a difference by race after 2 months. Receipt of first‐line therapy and the median number of standard‐guidelines therapies did not significantly differ by race/ethnicity.

**Implications:**

Our findings suggest that there is a modest difference in T2FT by race/ethnicity in the initial time period following diagnosis of mPDAC, but no reported difference in overall survival. Additional studies using a larger, more diverse cohort of patients are recommended to better understand the effects of race/ethnicity on the treatment and survival of patients with mPDAC.

## 1 Introduction

In 2024, it is estimated that pancreatic cancer will account for 3.3% of all new cancer diagnoses and for 8.5% of all cancer deaths^1^. The 5‐year survival rate for pancreatic adenocarcinoma (PDAC) is 12.5%, with the majority of PDAC patients diagnosed with metastatic disease [1]. PDAC 5‐year survival improves to 44.3% if diagnosed with localized disease, due to the option for curative surgery in this setting [2]. When compared to White patients, patients of color are more likely to be diagnosed with advanced stage disease [3]. Black patients are also less likely to undergo surgical resection for early stage PDAC, receive adjuvant chemotherapy or treatment for advanced disease, and use hospice services for end‐of‐life support compared to any other racial/ethnic group [3, 4, 5, 6, 7, 8]. These differences in stage at diagnosis and treatment received by race suggest that improvements in diagnosis and treatment outcomes could be made for patients with PDAC [9].

It is important to acknowledge that race is a social construct rather than a biological or genetic classification. Noted differences in treatment outcomes by race/ethnicity are often related to social determinants of health. Social determinants of health can be defined in terms of socioeconomic status (SES) and access to care. SES is often determined by an individual's education, income, and employment [10]. Access to care is often measured in terms of insurance status, but it is also influenced by the SES of an individual, such as whether they are employed. In the literature, differences in access to care and SES are noted by race/ethnicity [9], thus, racial and ethnic categorizations are widely used in medical research looking at differences in clinic outcomes due to associations with SES, access to care, and systemic inequities [10].

Access to care can also be measured in terms T2FT, which is an indicator of treatment delays, as well as the receipt of guideline concordant treatment [11, 12]. Patients with insurance are more likely to have access to care, seek treatment, be diagnosed at earlier stages, and undergo guidance‐concordant treatment for cancer compared to the uninsured [13, 14]. Compared to White patients, Black and Hispanic patients are more likely to be uninsured or under‐insured [15].

In addition to T2FT, disparities may be evident in the receipt of guidance‐concordant treatment for PDAC. Data is lacking regarding the race/ethnic differences in the type of chemotherapy regimens used for the treatment of mPDAC and the adherence to guideline‐concordant care. Thus, we sought to evaluate T2FT, receipt of standard guideline‐concordant first‐line chemotherapy, and overall survival in an mPDAC population by race/ethnicity using a large, real‐world multilevel dataset from the U.S. that includes SES, access, and clinical variables.

## 2 Materials and Methods

### 2.1 Study Population

We utilized the Flatiron Health electronic health record derived, deidentified database. This is a longitudinal, geographically, and demographically diverse U.S. database that contains normalized, aggregated, and harmonized patient‐level structure and unstructured data that is obtained via technology enabled chart abstraction [16, 17]. The Flatiron database includes over 250 community and academic cancer clinics, representing more than 2 million patients across the United States. mPDAC (ICD‐10‐CM C25) refers to cancer that originated, but spread outside the pancreas [17]. Patients diagnosed with mPDAC treated between 1/1/2014 and 5/31/2020 who presented with stage IV or recurrent metastatic disease were included in these analyses. Cases with a histologic diagnosis other than mPDAC, with unknown race/ethnicity, insurance status, stage at diagnosis, or gender were excluded. This study focused primarily on non‐Hispanic White (NHW), Hispanic, and non‐Hispanic Black (NHB) patients who were metastatic at initial diagnosis. This study used deidentified data and was determined to be nonhuman subjects' research by the local Institutional Review Board.

### 2.2 Clinical and Demographic Variables

Patient characteristics including age (continuous), gender (male, female), race, NHW, NHB, Hispanic, insurance type (commercial, Medicare, Medicaid, Medicare/commercial, no insurance, unknown), prior surgery (yes/no) and tobacco use (yes/no) were obtained. Disease information included stage at diagnosis, primary site (body, head, tail, overlapping sites, pancreas NOS), and ECOG performance status (PS) at treatment initiation. We also examined body mass index (BMI‐kg/m2) and eGFR at the time of diagnosis. Covariates with multiple values over time were defined based on their value at the time closest to diagnosis (within a window of 90 days).

### 2.3 Outcome

For this study, T2FT was defined as the difference between the date of diagnosis of metastatic disease and the date of initiation of first‐line systemic anticancer therapy in days. The primary objective of this study was to evaluate treatment differences by race/ethnicity in mPDAC patients. Receipt of standard‐guideline concordant first‐line therapy (yes/no) was defined based on NCCN guidelines recommendations [18]. Using NCCN recommendations, we categorized each first‐line treatment regimen into one of five categories: (1) Approved standard of care, (1a) generally acceptable modification, (2) treatment that could be appropriate depending on patient circumstances, (3) Clinical study drug, (4) unclear indication, and (5) No anti‐mPDAC treatment (See Table 3 for treatment regimen assignments by categories). Standard regimens, considered concordant with current treatment guidelines, included categories 1 and 1a and typically included gemcitabine alone, gemcitabine with nab‐paclitaxel, and FOLFIRINOX (5‐Fluorouracil, Oxaliplatin and Irinotecan) [18, 19, 20]. For the overall survival outcome (OS), patients who died or had not received any treatment at the end of follow‐up were considered censored at the date of death or last confirmed activity (e.g., physician visit).

### 2.4 Statistical Analysis

All analyses were conducted separately in the de novo metastatic (presented here) and recurrent metastatic populations (Appendix S1). We focused on the de novo metastatic findings in the main manuscript based on the fact that these patients would have barriers to access to care compared to the recurrent population who are already receiving care. Patient and clinical variables of interest were summarized by race/ethnic group and compared using Chi‐squared and *T*‐tests. Time until treatment was analyzed using survival methods for time‐to‐event data, where the start of systemic treatment was considered the event of interest. In univariate analyses, Kaplan–Meier curves with log‐rank tests were used to determine the association between race/ethnicity and time until treatment. We present cumulative incidence curves (1 minus the Kaplan–Meier estimate) to demonstrate the cumulative distribution of time until treatment. Next, we fit a Cox proportional hazards regression model to determine whether time until treatment differed by race. We assessed the proportional hazards assumption using complementary log–log plots and tests of Schoenfeld residuals. Where nonproportionality existed, we then fit Cox models with time‐varying effects of race. We used piecewise constant effects, allowing for a change in effects at 2 months; this cut point was informed by examining the cumulative incidence curves. Some covariates have missing data (see Table 1); we used multiple imputation via chained equations for covariates with < 35% missing values [21]. We created 5 imputed datasets and combined results via Rubin's rule. Associations between race/ethnicity and type of first‐line treatment for metastatic disease were tested via Chi‐squared tests. We defined standard treatment as categories 1 or 1a and used logistic regression models to determine the association between race and receipt of standard treatment, controlling for covariates of interest. As above, multiple imputation methods were used to handle missing data. Finally, we assessed disparities in overall survival by race using Kaplan–Meier curves with log‐rank tests and Cox proportional hazards regression models. These regression models included adjusting for covariates.

**TABLE 1:** Patient characteristics at diagnosis of metastatic disease.

Characteristics				Overall (*n* = 4471)	
	White (*N* = 3678)	Black (*N* = 530)	Hispanic (*N* = 263)	Total (*N* = 4471)	*p*
Gender					0.718
Female	1645 (44.7%)	247 (46.6%)	118 (44.9%)	2010 (45.0%)	
Male	2033 (55.3%)	283 (53.4%)	145 (55.1%)	2461 (55.0%)	
Age (years)					< 0.001
*N*	3678	530	263	4471	
Median	69.436	66.872	66.105	68.945	
Range	22.119–84.600	28.227–84.578	33.947–84.131	22.119–84.600	
Group stage					< 0.001
IV	3678 (100.0%)	530 (100.0%)	263 (100.0%)	4471 (100.0%)	
Smoking status					< 0.001
History of smoking	2131 (57.9%)	309 (58.3%)	109 (41.4%)	2549 (57.0%)	
No history of smoking	1537 (41.8%)	220 (41.5%)	153 (58.2%)	1910 (42.7%)	
Unknown/Not documented	10 (0.3%)	1 (0.2%)	1 (0.4%)	12 (0.3%)	
Primary Site					0.081
Body	788 (21.4%)	83 (15.7%)	56 (21.3%)	927 (20.7%)	
Head	1472 (40.0%)	209 (39.4%)	96 (36.5%)	1777 (39.7%)	
Overlapping sites	408 (11.1%)	68 (12.8%)	35 (13.3%)	511 (11.4%)	
Pancreas, NOS	172 (4.7%)	30 (5.7%)	14 (5.3%)	216 (4.8%)	
Tail	838 (22.8%)	140 (26.4%)	62 (23.6%)	1040 (23.3%)	
Receipt of surgery					0.223
No/unknown	3625 (98.6%)	524 (98.9%)	256 (97.3%)	4405 (98.5%)	
Yes	53 (1.4%)	6 (1.1%)	7 (2.7%)	66 (1.5%)	
BRCA positive					0.524
N‐Miss	3631	524	259	4414	
FALSE	36 (76.6%)	5 (83.3%)	4 (100.0%)	45 (78.9%)	
TRUE	11 (23.4%)	1 (16.7%)	0 (0.0%)	12 (21.1%)	
ca199 cleaned					0.257
*N*	2764	375	172	3311	
Median	1895.200	353.000	1859.500	1727.000	
Range	0.900–1611488.600	0.700–641100.000	0.900–2153552.000	0.700–2153552.000	
Year of metastatic diagnosis					0.165
2014	426 (11.6%)	50 (9.4%)	23 (8.7%)	499 (11.2%)	
2015	514 (14.0%)	63 (11.9%)	49 (18.6%)	626 (14.0%)	
2016	521 (14.2%)	62 (11.7%)	40 (15.2%)	623 (13.9%)	
2017	583 (15.9%)	83 (15.7%)	41 (15.6%)	707 (15.8%)	
2018	590 (16.0%)	97 (18.3%)	39 (14.8%)	726 (16.2%)	
2019	498 (13.5%)	79 (14.9%)	38 (14.4%)	615 (13.8%)	
2020	470 (12.8%)	85 (16.0%)	29 (11.0%)	584 (13.1%)	
2021	76 (2.1%)	11 (2.1%)	4 (1.5%)	91 (2.0%)	
Insurance type					< 0.001
Commercial	1494 (40.6%)	250 (47.2%)	119 (45.2%)	1863 (41.7%)	
Medicaid	149 (4.1%)	62 (11.7%)	27 (10.3%)	238 (5.3%)	
Medicare	774 (21.0%)	72 (13.6%)	47 (17.9%)	893 (20.0%)	
Medicare/Commercial	813 (22.1%)	77 (14.5%)	16 (6.1%)	906 (20.3%)	
Other	448 (12.2%)	69 (13.0%)	54 (20.5%)	571 (12.8%)	
ECOG score					0.019
N‐Miss	1093	152	97	1342	
0	947 (36.6%)	103 (27.2%)	65 (39.2%)	1115 (35.6%)	
1	1133 (43.8%)	180 (47.6%)	63 (38.0%)	1376 (44.0%)	
2	394 (15.2%)	75 (19.8%)	28 (16.9%)	497 (15.9%)	
3	105 (4.1%)	18 (4.8%)	9 (5.4%)	132 (4.2%)	
4	6 (0.2%)	2 (0.5%)	1 (0.6%)	9 (0.3%)	
BMI (categorical)					< 0.001
[0, 18.5]	139 (3.8%)	24 (4.5%)	9 (3.4%)	172 (3.8%)	
[18.5, 25]	1312 (35.7%)	186 (35.1%)	99 (37.6%)	1597 (35.7%)	
[25, 30]	1218 (33.1%)	155 (29.2%)	100 (38.0%)	1473 (32.9%)	
[30, 35]	611 (16.6%)	86 (16.2%)	32 (12.2%)	729 (16.3%)	
[35, 100]	345 (9.4%)	57 (10.8%)	20 (7.6%)	422 (9.4%)	
Unknown	53 (1.4%)	22 (4.2%)	3 (1.1%)	78 (1.7%)	
Time until treatment (months)					0.314
*N*	2863	399	200	3462	
Median	0.690	0.789	0.821	0.690	
eGFR					< 0.001
*N*	3457	497	218	4172	
Median	84.229	91.066	91.840	85.146	
Low eGFR					< 0.001
No	3204 (87.1%)	451 (85.1%)	204 (77.6%)	3859 (86.3%)	
Yes	253 (6.9%)	46 (8.7%)	14 (5.3%)	313 (7.0%)	
Unknown	221 (6.0%)	33 (6.2%)	45 (17.1%)	299 (6.7%)	
History of diabetes mellitus					< 0.001
No	3084 (83.8%)	403 (76.0%)	217 (82.5%)	3704 (82.8%)	
Yes	594 (16.2%)	127 (24.0%)	46 (17.5%)	767 (17.2%)	
Known diabetes mellitus					0.190
False	3462 (94.1%)	490 (92.5%)	251 (95.4%)	4203 (94.0%)	
True	216 (5.9%)	40 (7.5%)	12 (4.6%)	268 (6.0%)	

## 3 Results

The total study population included 6470 patients, of whom 4471 were metastatic at initial diagnosis and 1999 had recurrent metastatic disease. Recurrent metastatic population findings are included in Appendix S1. The primary study population of interest included the 4471 patients with an initial diagnosis of mPDAC. Table 1 shows a summary of the patient‐level and clinical variables that were considered in this analysis. More than 80% of patients were NHW, 11.8% NHB, and 5.9% Hispanic. Patient diagnosis age for metastatic disease (stage IV) ranged from 22 to 85 years, with just under half the patients having a primary malignancy of the head of the pancreas (39.7%). Median age at metastatic diagnosis was significantly different among the race/ethnic groups, with NHW (69.4 years) patients having been diagnosed 3 years later than NHB (66.9 years) and Hispanic (66.1 years) patients (*p* < 0.001).

Over half of the total patient population had a history of smoking (57%). NHW patients (57.9%) and NHB patients (58.3%) had a higher proportion of individuals with a history of smoking as compared to Hispanic patients (41.4%) (*p* < 0.001). For BMI (classified categorically), there was a significant difference among the populations within the overweight (BMI 25–30) range, as 38% of Hispanic patients were classified in this category, compared to 29.2% of NHB patients and 33.1% of NHW patients (*p* < 0.001). NHB (11.7%) and Hispanic patients (10.3%) were more likely than NHW patients (4.1%) to have Medicaid (*p* < 0.001). NHW patients (22.1%) also had more Medicare/Commercial insurance type, as compared to NHB (14.5%) and Hispanic patients (6.1%). ECOG PS was significantly different among the groups, as NHB and NHW patients had a majority ECOG score of 1, while Hispanic patients were more commonly ECOG PS of 0. When looking at ECOG PS > 2, NHB patients had the highest proportion at 25.1%, with the next highest being Hispanic patients at 22.9% and NHW at 19.5% (Table 1).

**FIGURE 1:**
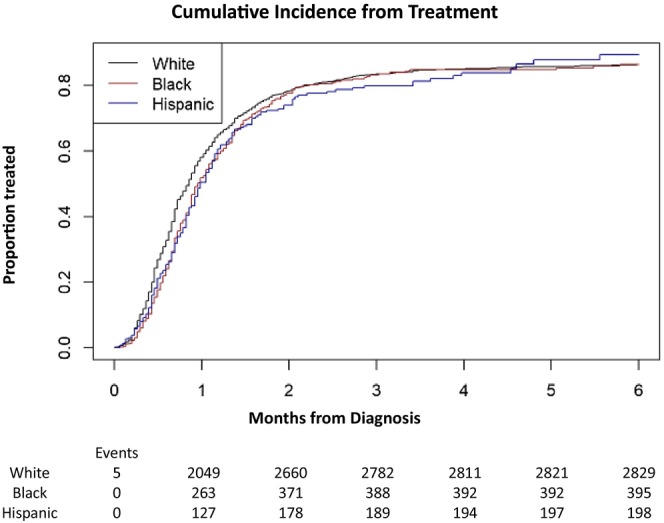
Cumulative incidence of treatment over time by race (*p* = 0.02).

There was a difference in T2FT for NHB compared to NHW and Hispanic patients (Figure 1). Median time to treatment was 25.7 days for NHW, 29.0 for NHB, and 30.0 for Hispanic patients (*p* = 0.02). In multivariable adjusted models, differences in T2FT by race/ethnicity persisted (Table 2). NHB patients were found to have longer time to first treatment [HR = 0.87 (95% CI: 0.78–0.974); *p* = 0.015] compared to White patients. The following patient variables also had an association with longer T2FT, including increasing age [HR = 0.96 (95% CI: 0.93–0.982); *p* = 0.001], primary malignancy site at the head of the pancreas [HR = 0.88 (95% CI: 0.81–0.967); *p* = 0.008], ECOG score of 2 [HR = 0.88 (95% CI: 0.78–0.984); *p* = 0.026], and ECOG PS 3 or 4 [HR = 0.70 (95% CI: 0.53–0.913); *p* = 0.018]. Higher BMI was associated with longer T2FT [HR = 1.01 (95% CI = 1.0–1.01); *p* = 0.04]. Male gender [HR = 1.1 (95% CI: 1.02–1.174); *p* = 0.009] and eGFR [HR = 1.00 (95% CI: 1–1.004); *p* = 0.024] were also associated with T2FT (see Table 2). The remaining variables were not statistically significant. We found some evidence of nonproportional hazards, likely because most patients were treated by 2 months. In the time‐varying models, we confirmed the significant association of race and time to treatment prior to 2 months. After 2 months, the effect of NHB race was attenuated and no longer significant (*p* = 0.25).

**TABLE 2:** Association of patient level and clinical variables for metastatic patients with time to first treatment.

Variables	HR	95% CI	*p*
Combined race/Ethnicity			
Black	0.87	0.78–0.974	**0.015**
Hispanic	0.88	0.76–1.018	0.086
Other	NA	NA	NA
Insurance type			
Medicaid	0.95	0.81–1.107	0.5
Medicare	0.95	0.86–1.046	0.291
Medicare/Commercial	1.03	0.93–1.134	0.569
Other	0.92	0.82–1.021	0.114
Male gender	1.1	1.02–1.174	0.009
Age10	1.65	1.16–2.354	0.006
*I* (Age10^2^)	0.96	0.93–0.982	**0.001**
No history of smoking	1	0.93–1.068	0.915
Primary site of mPDAC			
Head	0.88	0.81–0.967	**0.008**
Overlapping sites	0.93	0.82–1.051	0.242
Pancreas, NOS	0.87	0.73–1.033	0.112
Tail	0.92	0.83–1.021	0.118
Year at metastatic diagnosis			
2015	0.99	0.87–1.134	0.9
2016	0.97	0.85–1.107	0.631
2017	1.07	0.94–1.220	0.303
2018	0.91	0.8–1.041	0.173
2019	1.08	0.94–1.239	0.257
2020	1.07	0.93–1.227	0.346
2021	1.27	0.98–1.634	0.066
ECOG score 1	1.00	0.91–1.091	0.958
ECOG score 2	0.88	0.78–0.984	**0.026**
ECOG score 3–4	0.70	0.53–0.913	**0.018**
BMI	1.01	1–1.011	**0.043**
eGFR	1.00	1–1.004	**0.024**
History of diabetes mellitus	0.98	0.9–1.077	0.701

*Note:* Bold values indicate significance level 0.05.

Regimens used for first‐line treatments were categorized into 1 of 5 categories, where category 1 and 1a were considered guideline concordant (Table 3) and summarized by race/ethnicity (Table 4). There were significant differences in first‐line therapies by race/ethnicity. This could be driven by clinical trial participation of NHB (4%) and NHW patients (5.3%) that were more likely to enroll in clinical trials compared to Hispanic patients (1.9%). However, receipt of standard, guideline concordant therapy was very similar across race/ethnic groups. In addition, there was no significant difference in the median number of lines of therapy by race/ethnicity.

**TABLE 3:** First line treatment categories using NCCN recommendations.

1	Approved standard of care (1a = generally accepted modification)
2	Could be appropriate depending on patient circumstances
3	Clinical study drug
4	Unclear indication
5	No antipancreatic cancer treatment

**TABLE 4:** Summary of treatment categories for non‐Hispanic White versus non‐Hispanic Black versus Hispanic mPDAC patients.

Characteristics				Overall (*n* = 4471)	
	White (*N* = 3678)	Black (*N* = 530)	Hispanic (*N* = 263)	Total (*N* = 4471)	*p*
First line treatment category					0.160
1	2176 (59.2%)	303 (57.2%)	157 (59.7%)	2636 (59.0%)	
2	424 (11.5%)	64 (12.1%)	29 (11.0%)	517 (11.6%)	
3	194 (5.3%)	21 (4.0%)	5 (1.9%)	220 (4.9%)	
4	61 (1.7%)	9 (1.7%)	8 (3.0%)	78 (1.7%)	
5	823 (22.4%)	133 (25.1%)	64 (24.3%)	1020 (22.8%)	
L1Category 2					0.074
1	2176 (59.2%)	303 (57.2%)	157 (59.7%)	2636 (59.0%)	
1a	336 (9.1%)	53 (10.0%)	28 (10.6%)	417 (9.3%)	
2	88 (2.4%)	11 (2.1%)	1 (0.4%)	100 (2.2%)	
3	194 (5.3%)	21 (4.0%)	5 (1.9%)	220 (4.9%)	
4	61 (1.7%)	9 (1.7%)	8 (3.0%)	78 (1.7%)	
5	823 (22.4%)	133 (25.1%)	64 (24.3%)	1020 (22.8%)	
Total lines of treatment					0.223
*N*	3678	530	263	4471	
Median	1.000	1.000	1.000	1.000	
Range	0.000–9.000	0.000–5.000	0.000–6.000	0.000–9.000	
Total lines of treatment (Categorical)					0.806
0	815 (22.2%)	131 (24.7%)	63 (24.0%)	1009 (22.6%)	
1	1685 (45.8%)	237 (44.7%)	118 (44.9%)	2040 (45.6%)	
2	736 (20.0%)	107 (20.2%)	52 (19.8%)	895 (20.0%)	
3	306 (8.3%)	40 (7.5%)	24 (9.1%)	370 (8.3%)	
4+	136 (3.7%)	15 (2.8%)	6 (2.3%)	157 (3.5%)	

**FIGURE 2:**
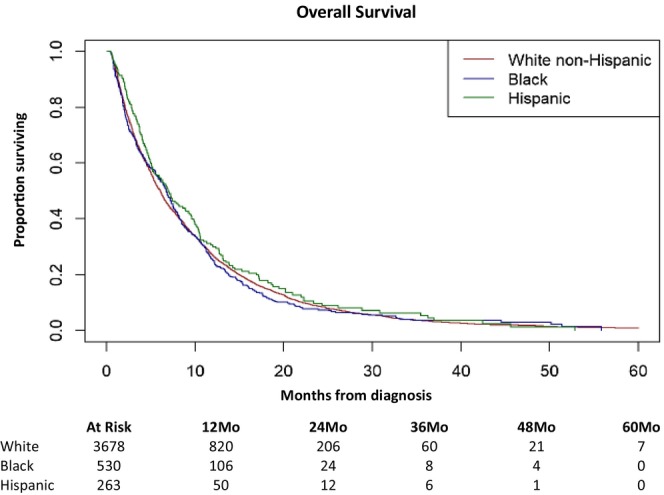
Overall survival by race/ethnicity in patients with metastatic disease at diagnosis.

In multivariable analysis, no statistically significant differences were found in standard, guideline‐concordant therapy between NHB (OR = 0.94; *p = 0.57*) and Hispanic (OR = 1.09; *p = 0.556*) patients versus NHW patients (Table 5). However, patients with an ECOG PS of 2 or 3–4 were significantly less likely to have received guidance concordance first‐line treatment (OR = 0.77, *p* = 0.024; OR = 0.40, *p* = 0.002). No statistical differences were noted in OS by race and Hispanic ethnicity (*p* = 0.30) (Figure 2) These results were maintained after adjusting for covariates. As expected, patient factors such as older age and higher ECOG PS were associated with decreased OS.

**TABLE 5:** Multivariable regression analysis predicting receipt of standard first‐line treatment in patients metastatic at diagnosis.

Characteristics	OR	95% CI	*p*
Combined race/Ethnicity			
Black	0.94	0.77–1.157	0.57
Hispanic	1.09	0.82–1.45	0.556
Insurance type			
Medicaid	0.98	0.72–1.335	0.899
Medicare	0.95	0.79–1.14	0.572
Medicare/Commercial	1.08	0.9–1.303	0.408
Other	0.92	0.75–1.138	0.455
Male	1.00	0.87–1.136	0.95
Age10	0.91	0.83–0.988	0.025
Smoking status			
No history of smoking	0.88	0.77–1.008	0.065
Primary site			
Head	1.08	0.91–1.294	0.375
Overlapping sites	1.08	0.85–1.375	0.531
Pancreas, NOS	0.79	0.57–1.085	0.145
Tail	0.87	0.71–1.055	0.154
Surgery yes	0.65	0.39–1.075	0.093
Year at metastatic diagnosis			
2015	1.09	0.84–1.418	0.509
2016	0.96	0.74–1.245	0.767
2017	1.13	0.87–1.455	0.365
2018	0.85	0.66–1.098	0.219
2019	1.13	0.87–1.472	0.369
2020	1.08	0.82–1.404	0.591
2021	1.14	0.69–1.883	0.617
ECOG score 1	0.95	0.78–1.163	0.629
ECOG score 2	0.77	0.62–0.963	**0.024**
ECOG score 3–4	0.4	0.25–0.622	**0.002**
BMI	1.01	1–1.025	0.021
eGFR	1.01	1–1.01	0.001
Bilirubin	0.95	0.94–0.97	< 0.001
History of diabetes mellitus	0.94	0.79–1.114	0.459

*Note:* Bold values indicate significance level 0.05.

## 4 Discussion

This study addresses existing research gaps and provides insights into treatment‐related disparities across different race/ethnic groups including NHB, NHW, and Hispanic patients with mPDAC. We found that NHB patients were more likely than NHW patients to have longer T2FT, particularly within the first 2 months after diagnosis. However, no race/ethnic differences were reported between NHB, NHW, and Hispanic patients for OS. Further, race/ethnic differences were not observed in the receipt of guideline‐concordant therapy, though Hispanic patients were less likely to participate in clinical trials compared to NHW or NHB patients.

Differences in T2FT between NHB and NHW patients persisted with and without adjustments for clinical covariates. This suggests that there are unexplained covariates contributing to race/ethnic differences in T2FT that we were unable to measure in our study. These relate to social determinants of health, including, but not limited to systemic racism and subsequent medical mistrust, economic stability, education levels, general access to healthcare, as well as neighborhood environment. A recent study concluded that patients from “resource‐poor neighborhoods” are generally less likely to receive “stage appropriate” therapy for pancreatic cancer, which includes the period from initial diagnosis until first treatment decisions are made [22]. Disparities that exist in not only the diagnosis, but also the treatment, of pancreatic cancer are largely driven by race/ethnicity and SES, of which insurance is a major factor [23]. The effects of various social determinants of health impact nearly every aspect of pancreatic cancer treatment, making it difficult in our study to discern the effect of race/ethnicity alone on time to treatment initiation.

However, no race/ethnic differences in survival were noted in this study, despite differences in time to first treatment between NHB and NHW patients. Potential explanations include that the study population was primarily White and insured, which could make evaluation of effects in small populations of underserved patients difficult to detect. The severity of metastatic disease could override effects of time to first treatment; given clinical variables remained significantly associated with survival, this could be because there is no strictly accepted and followed threshold for ideal time of treatment initiation of mPDAC. While the acuity of when treatment occurs is still important, it may be outweighed by the aggressive nature of metastatic pancreatic cancer [24]. Factors that influence time until treatment initiation may include the necessity for repeated biopsies, registration in a clinical trial, or transfer to a special cancer treatment center, all of which do not have a strong adverse relationship with treatment outcome [25]. Thus, our findings suggest that time to first treatment may potentially be an indicator of quality of care in this population, but not necessarily survival.

Patients who did not receive standard treatments (categories 2–5) were considered nonconcordant. This study is one of the first to evaluate differences in rates of receiving standard (approved, guideline compliant) first‐line therapies by race/ethnicity, including detailed therapy regimens by agent, time on treatment, and number of lines of therapy. Treatment‐related variables, like time to first treatment, serving as a quality‐of‐care indicator, are also further supported by the finding that overall, guideline‐concordant care was not different by race/ethnicity, except for Hispanics being less likely to receive a clinical study drug than NHW or NHB patients. Other barriers to care, including language barriers [26], that were not measured in this study could be contributing to potential treatment differences by race/ethnicity for Hispanic patients. Specifically, the lack of written trial information in a native language, which is part of the larger set of unique socio‐cultural contexts that affects certain minority populations' ability to participate in clinical trial [27]. Notably, the difference in time to first treatment was only significant during the first 2 months after diagnosis, so factors leading to long delays may have been different than drivers of delays in the initial period following diagnosis. Nevertheless, our analysis did demonstrate an association between race and time to treatment during the initial 2 months after diagnosis, when the majority of patients initiate treatment.

Certain clinical variables, like increasing age, were significantly associated with a longer time to first treatment. Other significant associations included primary cancer site at the head of the pancreas as well as ECOG PS > 2. All these factors indicate a sicker patient, which may in part explain the delay in treatment. It is possible that the course of treatment is more complex or that there are more comorbid barriers to starting treatment, resulting in a prolonged T2FT. This is reinforced when looking at OS, as age and worsening ECOG PS are significantly associated with shorter survival times [28]. We did not find associations between race in patients with recurrent metastatic disease (see Appendix S1). We theorize that this is because recurrent patients have already established relationships with the healthcare system, so barriers in accessing care will be lessened.

A limitation of this study is that the Flatiron Health dataset is not a representative sample of U.S. cancer cases, particularly with regard to the representation of Black patients. Due to the limited sample size of Black patients, it was not feasible to perform a matched or stratified analysis. Consequently, the model estimates presented here may not be fully generalizable to Black patients, and caution should be exercised when extrapolating the findings to this group. The patients are ascertained from private practice oncology clinics and tertiary care centers; patients seen at safety‐net hospitals are not included in this dataset. Further, as in any observational study, there may be biases due to unmeasured confounding. Factors such as income, marital status, and comorbidity score are not available for analysis, and omission of such variables could bias findings. Data are also subject to misclassification or missingness; although we used multiple imputation methods to deal with missing data in our analysis, this relies on the assumption that the data are missing at random. Finally, it is important to note that the race and ethnicity data used in this study were self‐reported, reflecting individuals' social identities rather than genetic ancestry. Self‐reported race is influenced by sociopolitical and cultural factors, which shape healthcare access, provider biases, and treatment decisions [29]. This distinction is critical, as race is a social construct that does not correspond to underlying genetic differences but rather to lived experiences and systemic inequities [29]. In contrast, genetic ancestry reflects inherited genetic variation and does not inherently drive disparities in healthcare outcomes. Understanding these distinctions is essential for interpreting racial disparities in medical research and underscores the need for policies that address the structural and societal factors contributing to inequities in cancer treatment and outcomes.

## 5 Conclusion

Real‐world evidence plays a critical role in identifying and addressing disparities in cancer treatment. By capturing data from diverse patient populations, it offers insights that complement findings from clinical trials, which often have strict eligibility criteria and may underrepresent certain racial and ethnic groups. These real‐world insights can inform policy initiatives at the national, as well as hospital level, aimed at reducing disparities by improving access to timely and guideline‐concordant therapies for patients. Efforts such as expanded insurance coverage, patient navigation programs, and enhanced provider education in hospitals for patient groups with differences in health outcomes may help mitigate delays in treatment initiation and ensure equitable care delivery for patients with metastatic pancreatic cancer.

In this study, there was a suggestion that there are differences in T2FT as well as receipt of standard treatment protocols, and overall survival by race/ethnicity, both unadjusted and adjusted for additional SES variables (e.g., insurance status) as well as clinical covariates (age, gender, stage at diagnosis, performance status, and tobacco use). Our results demonstrated that NHB patients were more likely than NHW patients to have a longer time to first treatment, specifically in the 2‐month period following initial diagnosis. However, there is no reported difference in OS among the different race/ethnic groups. Further research using a larger, more diverse cohort of patients is warranted to examine how treatment‐related variables, like time to first treatment, and outcome variables, such as overall survival, differ among race/ethnic groups.

## Author Contributions

**Lavanya Aluri:** writing – original draft (lead), writing – review and editing (equal). **Christina Brown:** writing – original draft (lead), writing – review and editing (equal). **Anush Sridharan:** investigation (equal), supervision (equal), writing – review and editing (equal). **Elizabeth Handorf:** formal analysis (lead), methodology (supporting), software (equal), writing – review and editing (equal). **Kristen Sorice:** data curation (lead), project administration (lead), resources (lead), writing – original draft (supporting), writing – review and editing (equal). **Efrat Dotan:** conceptualization (supporting), funding acquisition (lead), methodology (supporting), writing – review and editing (equal). **Shannon M. Lynch:** conceptualization (lead), funding acquisition (lead), methodology (lead), writing – original draft (equal), writing – review and editing (equal).

## Ethics Statement

This study used deidentified data and was determined to be nonhuman subject's research by the local Institutional Review Board.

## Conflicts of Interest

The authors declare no conflicts of interest.

## Supporting information


**Appendix S1:** cam471108‐sup‐0001‐AppendixS1.docx.

## Data Availability

Data cannot be shared for the purpose of maintaining ethical boundaries and protecting the privacy of the patients involved in this research. There is summary level data available within this manuscript.
