# Supplementary material for: Investigation of Treatment‐Related Disparities in Metastatic Pancreatic Cancer Patients Using Real‐World Data
Source: Cancer Med. 2025 Aug 7;14(15):e71108. doi: 10.1002/cam4.71108 (PMC12329563; doi:10.1002/cam4.71108)
Supplement: Supplementary file 1 — Appendix S1: cam471108‐sup‐0001‐AppendixS1.docx. [file CAM4-14-e71108-s001.docx]

**Appendix S1**

| **Table 1.** Patient characteristics at diagnosis of recurrent metastatic disease | | | | | |
| --- | --- | --- | --- | --- | --- |
| Characteristics | | | | Overall (n=1999) | |
|  | **White (N=1693)** | **Black (N=199)** | **Hispanic (N=107)** | **Total (N=1999)** | **P value** |
| Gender |  |  |  |  |  |
| Female | 810 (47.8%) | 117 (58.8%) | 54 (50.5%) | 981 (49.1%) |  |
| Male | 883 (52.2%) | 82 (41.2%) | 53 (49.5%) | 1018 (50.9%) |  |
| Age (years) |  |  |  |  | <0.001 |
| N | 1693 | 199 | 107 | 1999 |  |
| Median | 69.875 | 67.31 | 68.227 | 69.572 |  |
| Range | 32.772 - 84.643 | 27.915 - 84.307 | 41.161 - 84.090 | 27.915 - 84.643 |  |
| Group Stage |  |  |  |  | <0.001 |
| I | 17 (1.0%) | 4 (2.0%) | 0 (0.0%) | 21 (1.1%) |  |
| IA | 51 (3.0%) | 8 (4.0%) | 4 (3.7%) | 63 (3.2%) |  |
| IB | 220 (13.0%) | 21 (10.6%) | 16 (15.0%) | 257 (12.9%) |  |
| II | 28 (1.7%) | 3 (1.5%) | 3 (2.8%) | 34 (1.7%) |  |
| IIA | 290 (17.1%) | 39 (19.6%) | 13 (12.1%) | 342 (17.1%) |  |
| IIB | 639 (37.7%) | 63 (31.7%) | 42 (39.3%) | 744 (37.2%) |  |
| III | 448 (26.5%) | 61 (30.7%) | 29 (27.1%) | 538 (26.9%) |  |
| Smoking Status |  |  |  |  | 0.304 |
| History of smoking | 960 (56.7%) | 109 (54.8%) | 50 (46.7%) | 1119 (56.0%) |  |
| No history of smoking | 730 (43.1%) | 90 (45.2%) | 57 (53.3%) | 877 (43.9%) |  |
| Unknown/not documented | 3 (0.2%) | 0 (0.0%) | 0 (0.0%) | 3 (0.2%) |  |
| Primary Site |  |  |  |  | 0.01 |
| Body | 245 (14.5%) | 25 (12.6%) | 9 (8.4%) | 279 (14.0%) |  |
| Head | 1195 (70.6%) | 142 (71.4%) | 82 (76.6%) | 1419 (71.0%) |  |
| Overlapping sites | 97 (5.7%) | 3 (1.5%) | 4 (3.7%) | 104 (5.2%) |  |
| Pancreas, NOS | 13 (0.8%) | 0 (0.0%) | 0 (0.0%) | 13 (0.7%) |  |
| Tail | 143 (8.4%) | 29 (14.6%) | 12 (11.2%) | 184 (9.2%) |  |
| Receipt of Surgery |  |  |  |  | 0.006 |
| No/unknown | 644 (38.0%) | 98 (49.2%) | 47 (43.9%) | 789 (39.5%) |  |
| Yes | 1049 (62.0%) | 101 (50.8%) | 60 (56.1%) | 1210 (60.5%) |  |
| BRCA Positive |  |  |  |  | 0.473 |
| N-Miss | 1416 | 169 | 87 | 1672 |  |
| FALSE | 261 (94.2%) | 29 (96.7%) | 20 (100.0%) | 310 (94.8%) |  |
| TRUE | 16 (5.8%) | 1 (3.3%) | 0 (0.0%) | 17 (5.2%) |  |
| ca199 Cleaned |  |  |  |  | 0.85 |
| N | 1375 | 152 | 88 | 1615 |  |
| Median | 256.3 | 186.5 | 172.75 | 247.1 |  |
| Range | 0.600 - 398532.300 | 1.000 - 237332.000 | 1.000 - 55232.970 | 0.600 - 398532.300 |  |
| Year of Metastatic Diagnosis |  |  |  |  | 0.445 |
| 2014 | 176 (10.4%) | 23 (11.6%) | 14 (13.1%) | 213 (10.7%) |  |
| 2015 | 207 (12.2%) | 12 (6.0%) | 11 (10.3%) | 230 (11.5%) |  |
| 2016 | 229 (13.5%) | 24 (12.1%) | 15 (14.0%) | 268 (13.4%) |  |
| 2017 | 277 (16.4%) | 25 (12.6%) | 18 (16.8%) | 320 (16.0%) |  |
| 2018 | 247 (14.6%) | 33 (16.6%) | 14 (13.1%) | 294 (14.7%) |  |
| 2019 | 256 (15.1%) | 38 (19.1%) | 15 (14.0%) | 309 (15.5%) |  |
| 2020 | 260 (15.4%) | 38 (19.1%) | 16 (15.0%) | 314 (15.7%) |  |
| 2021 | 41 (2.4%) | 6 (3.0%) | 4 (3.7%) | 51 (2.6%) |  |
| Insurance Type |  |  |  |  | <0.001 |
| Commercial | 628 (37.1%) | 80 (40.2%) | 39 (36.4%) | 747 (37.4%) |  |
| Medicaid | 54 (3.2%) | 25 (12.6%) | 11 (10.3%) | 90 (4.5%) |  |
| Medicare | 367 (21.7%) | 27 (13.6%) | 16 (15.0%) | 410 (20.5%) |  |
| Medicare/Commercial | 446 (26.3%) | 45 (22.6%) | 17 (15.9%) | 508 (25.4%) |  |
| Other | 198 (11.7%) | 22 (11.1%) | 24 (22.4%) | 244 (12.2%) |  |
| ECOG Score |  |  |  |  | 0.981 |
| N-Miss | 395 | 44 | 33 | 472 |  |
| 0 | 421 (32.4%) | 48 (31.0%) | 25 (33.8%) | 494 (32.4%) |  |
| 1 | 635 (48.9%) | 77 (49.7%) | 37 (50.0%) | 749 (49.1%) |  |
| 2 | 188 (14.5%) | 22 (14.2%) | 9 (12.2%) | 219 (14.3%) |  |
| 3 | 49 (3.8%) | 8 (5.2%) | 3 (4.1%) | 60 (3.9%) |  |
| 4 | 5 (0.4%) | 0 (0.0%) | 0 (0.0%) | 5 (0.3%) |  |
| BMI |  |  |  |  | 0.019 |
| N | 1638 | 190 | 106 | 1934 |  |
| Median | 23.678 | 23.948 | 22.853 | 23.657 |  |
| BMI (categorical) |  |  |  |  | 0.005 |
| [0,18.5) | 158 (9.3%) | 18 (9.0%) | 12 (11.2%) | 188 (9.4%) |  |
| [18.5,25) | 860 (50.8%) | 84 (42.2%) | 61 (57.0%) | 1005 (50.3%) |  |
| [25,30) | 417 (24.6%) | 47 (23.6%) | 20 (18.7%) | 484 (24.2%) |  |
| [30,35) | 152 (9.0%) | 24 (12.1%) | 10 (9.3%) | 186 (9.3%) |  |
| [35,100] | 51 (3.0%) | 17 (8.5%) | 2 (1.9%) | 70 (3.5%) |  |
| Unknown | 55 (3.2%) | 9 (4.5%) | 2 (1.9%) | 66 (3.3%) |  |
| Time Until Treatment (months) |  |  |  |  | 0.479 |
| N | 1184 | 143 | 80 | 1407 |  |
| Median | 0.657 | 0.69 | 0.575 | 0.657 |  |
| eGFR |  |  |  |  | < 0.001 |
| N | 1638 | 191 | 102 | 1931 |  |
| Median | 86.642 | 96.013 | 89.026 | 87.18 |  |
| Low eGFR |  |  |  |  | 0.533 |
| No | 1534 (90.6%) | 174 (87.4%) | 97 (90.7%) | 1805 (90.3%) |  |
| Yes | 104 (6.1%) | 17 (8.5%) | 5 (4.7%) | 126 (6.3%) |  |
| Unknown | 55 (3.2%) | 8 (4.0%) | 5 (4.7%) | 68 (3.4%) |  |
| Albumin |  |  |  |  | 0.243 |
| N | 1473 | 177 | 89 | 1739 |  |
| Median | 37 | 37 | 36 | 37 |  |
| Low Albumin |  |  |  |  | 0.713 |
| No | 1042 (61.5%) | 124 (62.3%) | 62 (57.9%) | 1228 (61.4%) |  |
| Yes | 431 (25.5%) | 53 (26.6%) | 27 (25.2%) | 511 (25.6%) |  |
| Unknown | 220 (13.0%) | 22 (11.1%) | 18 (16.8%) | 260 (13.0%) |  |
| Bilirubin |  |  |  |  | 0.263 |
| N | 1634 | 191 | 102 | 1927 |  |
| Median | 0.5 | 0.5 | 0.5 | 0.5 |  |
| High Bilirubin |  |  |  |  | 0.154 |
| No | 1523 (90.0%) | 181 (91.0%) | 89 (83.2%) | 1793 (89.7%) |  |
| Yes | 111 (6.6%) | 10 (5.0%) | 13 (12.1%) | 134 (6.7%) |  |
| Unknown | 59 (3.5%) | 8 (4.0%) | 5 (4.7%) | 72 (3.6%) |  |
| History of Diabetes Mellitus |  |  |  |  | 0.035 |
| No | 1379 (81.5%) | 151 (75.9%) | 79 (73.8%) | 1609 (80.5%) |  |
| Yes | 314 (18.5%) | 48 (24.1%) | 28 (26.2%) | 390 (19.5%) |  |
| Known Diabetes Mellitus |  |  |  |  | 0.535 |
| FALSE | 1520 (89.8%) | 181 (91.0%) | 93 (86.9%) | 1794 (89.7%) |  |
| TRUE | 173 (10.2%) | 18 (9.0%) | 14 (13.1%) | 205 (10.3%) |  |

| **Table 2** Association of patient level and clinical variables for recurrent metastatic patients with Time to First Treatment (continuous values) | | | | |
| --- | --- | --- | --- | --- |
| **Variables** | **HR** | **95% CI** | **P value** | |
| Combined Race/Ethnicity |  |  |  |  |
| Black | 0.97 | 0.81 - 1.161 | 0.734 |  |
| Hispanic | 1.2 | 0.96 - 1.516 | 0.116 |  |
| Other | NA | NA | NA |  |
| Insurance Type |  |  |  |  |
| Medicaid | 0.89 | 0.68 - 1.156 | 0.383 |  |
| Medicare | 0.98 | 0.84 - 1.144 | 0.778 |  |
| Medicare/Commercial | 0.93 | 0.81 - 1.079 | 0.349 |  |
| Other | 0.87 | 0.73 - 1.044 | 0.139 |  |
| Male | 1.04 | 0.93 - 1.159 | 0.496 |  |
| Age10 | 0.88 | 0.82 - 0.946 | 0.001 |  |
| I(Age10^2) |  |  |  |  |
| No history of smoking | 0.97 | 0.87 - 1.087 | 0.641 |  |
| IsSurgeryYes | 1.02 | 0.91 - 1.146 | 0.729 |  |
| Primary Site of mPDAC |  |  |  |  |
| Head | 0.85 | 0.73 - 0.992 | 0.039 |  |
| Overlapping sites | 0.84 | 0.64 - 1.105 | 0.218 |  |
| Pancreas, NOS | 0.77 | 0.39 - 1.512 | 0.446 |  |
| Tail | 0.79 | 0.63 - 0.993 | 0.043 |  |
| Year at Metastatic Diagnosis |  |  |  |  |
| 2015 | 0.98 | 0.78 - 1.243 | 0.884 |  |
| 2016 | 1.09 | 0.87 - 1.36 | 0.471 |  |
| 2017 | 1.14 | 0.92 - 1.412 | 0.228 |  |
| 2018 | 1.13 | 0.91 - 1.406 | 0.271 |  |
| 2019 | 1.16 | 0.93 - 1.434 | 0.188 |  |
| 2020 | 1.23 | 0.99 - 1.517 | 0.063 |  |
| 2021 | 1.07 | 0.73 - 1.59 | 0.721 |  |
| ECOG Score 1 | 0.98 | 0.86 - 1.118 | 0.768 |  |
| ECOG Score 2 | 0.89 | 0.72 - 1.093 | 0.267 |  |
| ECOG Score 3-4 | 0.32 | 0.19 - 0.535 | <0.001 |  |
| BMI | 1.01 | 1 - 1.017 | 0.233 |  |
| eGFR | 1 | 1 - 1.006 | 0.095 |  |
| bilirubin | 0.97 | 0.93 - 1.009 | 0.12 |  |
| History of Diabetes Mellitus | 1.04 | 0.91 - 1.189 | 0.586 |  |

**Table 3.** Summary of treatment categories for non-Hispanic White vs. non-Hispanic Black vs. Hispanic recurrent metastatic patients

| Characteristics | | | | Overall (n=1999) | |
| --- | --- | --- | --- | --- | --- |
|  | **White (N=1693)** | **Black (N=199)** | **Hispanic (N=107)** | **Total (N=1999)** | **P value** |
| L1Category 2 |  |  |  |  | 0.897 |
| 1 | 692 (40.9%) | 79 (39.7%) | 46 (43.0%) | 817 (40.9%) |  |
| 2 | 392 (23.2%) | 51 (25.6%) | 26 (24.3%) | 469 (23.5%) |  |
| 3 | 52 (3.1%) | 7 (3.5%) | 3 (2.8%) | 62 (3.1%) |  |
| 4 | 44 (2.6%) | 6 (3.0%) | 5 (4.7%) | 55 (2.8%) |  |
| 5 | 513 (30.3%) | 56 (28.1%) | 27 (25.2%) | 596 (29.8%) |  |
| L1Category 2 |  |  |  |  | 0.657 |
| 1 | 692 (40.9%) | 79 (39.7%) | 46 (43.0%) | 817 (40.9%) |  |
| 1a | 340 (20.1%) | 49 (24.6%) | 22 (20.6%) | 411 (20.6%) |  |
| 2 | 52 (3.1%) | 2 (1.0%) | 4 (3.7%) | 58 (2.9%) |  |
| 3 | 52 (3.1%) | 7 (3.5%) | 3 (2.8%) | 62 (3.1%) |  |
| 4 | 44 (2.6%) | 6 (3.0%) | 5 (4.7%) | 55 (2.8%) |  |
| 5 | 513 (30.3%) | 56 (28.1%) | 27 (25.2%) | 596 (29.8%) |  |
| Total Lines of Treatment |  |  |  |  | 0.929 |
| N | 1693 | 199 | 107 | 1999 |  |
| Median | 1 | 1 | 1 | 1 |  |
| Range | 0.000 - 7.000 | 0.000 - 7.000 | 0.000 - 4.000 | 0.000 - 7.000 |  |
| Total Lines of Treatment (Categorical) |  |  |  |  | 0.497 |
| 0 | 509 (30.1%) | 56 (28.1%) | 27 (25.2%) | 592 (29.6%) |  |
| 1 | 692 (40.9%) | 86 (43.2%) | 54 (50.5%) | 832 (41.6%) |  |
| 2 | 329 (19.4%) | 40 (20.1%) | 17 (15.9%) | 386 (19.3%) |  |
| 3 | 117 (6.9%) | 15 (7.5%) | 8 (7.5%) | 140 (7.0%) |  |
| 4+ | 46 (2.7%) | 2 (1.0%) | 1 (0.9%) | 49 (2.5%) |  |

**Table 4.** Multivariate analysis of non-metastatic patients in treatment categories with time to first treatment

| **Characteristics** | **OR** | **95% CI** | **P value** |
| --- | --- | --- | --- |
| Combined Race/Ethnicity |  |  |  |
| Black | 1.09 | 0.78 - 1.505 | 0.623 |
| Hispanic | 1.15 | 0.75 - 1.773 | 0.513 |
| Insurance Type |  | - |  |
| Medicaid | 1.09 | 0.67 - 1.768 | 0.741 |
| Medicare | 0.98 | 0.75 - 1.295 | 0.902 |
| Medicare/Commercial | 0.92 | 0.72 - 1.195 | 0.551 |
| Other | 0.97 | 0.71 - 1.33 | 0.86 |
| Male | 0.94 | 0.78 - 1.146 | 0.563 |
| Age10 | 0.91 | 0.8 - 1.031 | 0.135 |
| Smoking Status |  |  |  |
| No history of smoking | 0.97 | 0.8 - 1.18 | 0.774 |
| Primary Site |  |  |  |
| Head | 0.75 | 0.57 - 1.003 | 0.052 |
| Overlapping sites | 0.95 | 0.58 - 1.552 | 0.827 |
| Pancreas, NOS | 1.1 | 0.32 - 3.796 | 0.881 |
| Tail | 0.65 | 0.43 - 0.98 | 0.04 |
| Surgery Yes | 1.27 | 1.04 - 1.551 | 0.019 |
| Year at Metastatic Diagnosis |  |  |  |
| 2015 | 0.86 | 0.57 - 1.285 | 0.46 |
| 2016 | 0.96 | 0.65 - 1.426 | 0.855 |
| 2017 | 1.07 | 0.73 - 1.551 | 0.734 |
| 2018 | 0.99 | 0.68 - 1.452 | 0.977 |
| 2019 | 1.24 | 0.85 - 1.823 | 0.269 |
| 2020 | 1.32 | 0.9 - 1.927 | 0.158 |
| 2021 | 0.78 | 0.41 - 1.485 | 0.444 |
| ECOG Score 1 | 0.84 | 0.67 - 1.067 | 0.158 |
| ECOG Score 2 | 0.68 | 0.49 - 0.959 | 0.032 |
| ECOG Score 3-4 | 0.17 | 0.09 - 0.312 | <0.001 |
| BMI | 1.03 | 1.01 - 1.05 | 0.004 |
| eGFR | 1 | 1 - 1.01 | 0.064 |
| Bilirubin | 0.91 | 0.86 - 0.97 | 0.004 |
| History of Diabetes Mellitus | 1.17 | 0.92 - 1.498 | 0.208 |

| **Table 5.** Association of patient level and clinical variables for metastatic patients with Overall Survival | | | | |
| --- | --- | --- | --- | --- |
| **Variables** | **HR** | **95% CI** | **P value** | |
| Combined Race/Ethnicity |  |  |  |  |
| Black | 1.00 | 0.90 - 1.112 | 0.984 |  |
| Hispanic | 0.09 | 0.77 – 1.051 | 0.18 |  |
| Other | NA | NA | NA |  |
| Insurance Type |  |  |  |  |
| Medicaid | 1.00 | 0.86 - 1.165 | 0.981 |  |
| Medicare | 0.87 | 0.79 – 0.956 | 0.004 |  |
| Medicare/Commercial | 0.97 | 0.89 - 1.069 | 0.572 |  |
| Other | 0.94 | 0.94 - 1.048 | 0.276 |  |
| Male | 1.013 | 1.06 – 1.210 | <0.001 |  |
| Age10 | 0.55 | 0.39 - 0.775 | 0.001 |  |
| I(Age10^2) | 1.05 | 1.03 – 1.082 | <0.001 |  |
| No history of smoking | 0.97 | 0.91 - 1.039 | 0.403 |  |
| Primary Site of mPDAC |  |  |  |  |
| Head | 0.96 | 0.87 – 1.046 | 0.323 |  |
| Overlapping sites | 1.12 | 1.00 - 1.270 | 0.06 |  |
| Pancreas, NOS | 1.12 | 0.94 - 1.329 | 0.191 |  |
| Tail | 1.14 | 1.03 – 1.255 | 0.011 |  |
| Year at Metastatic Diagnosis |  |  |  |  |
| 2015 | 1.04 | 0.91 - 1.180 | 0.571 |  |
| 2016 | 1.08 | 0.95 - 1.230 | 0.217 |  |
| 2017 | 1.02 | 0.90 - 1.155 | 0.756 |  |
| 2018 | 1.08 | 0.96 - 1.221 | 0.219 |  |
| 2019 | 0.97 | 0.85 - 1.111 | 0.692 |  |
| 2020 | 1.03 | 0.90 - 1.187 | 0.639 |  |
| 2021 | 1.06 | 0.77 - 1.466 | 0.725 |  |
| ECOG Score 1 | 1.26 | 1.13 – 1.404 | 0.001 |  |
| ECOG Score 2 | 1.83 | 1.60 - 2.102 | <0.001 |  |
| ECOG Score 3-4 | 2.34 | 1.86 – 2.949 | <0.001 |  |
| BMI | 1.00 | 1 - 1.008 | 0.566 |  |
| eGFR | 1.00 | 0.99 – 0.998 | <0.001 |  |
| bilirubin | 1.03 | 1.02 - 1.043 | <0.001 |  |
| History of Diabetes Mellitus | 1.06 | 0.98 - 1.161 | 0.163 |  |
